# Comparative Assessment of Air Pollution–Related Health Risks in Houston

**DOI:** 10.1289/ehp.10043

**Published:** 2007-07-05

**Authors:** Ken Sexton, Stephen H. Linder, Dritana Marko, Heidi Bethel, Philip J. Lupo

**Affiliations:** 1 The University of Texas School of Public Health, Brownsville, Texas, USA; 2 The University of Texas School of Public Health, Institute for Health Policy, Houston, Texas, USA; 3 U.S. Environmental Protection Agency, Office of Water, Office of Science and Technology, Health and Ecological Criteria Division, Washington, DC, USA; 4 The University of Texas School of Public Health, Division of Epidemiology, Houston, Texas, USA

**Keywords:** air pollution, air toxics, comparative risk, diesel particles, hazardous pollutants, particulate matter, risk assessment

## Abstract

**Background:**

Airborne emissions from numerous point, area, and mobile sources, along with stagnant meteorologic conditions, contribute to frequent episodes of elevated air pollution in Houston, Texas. To address this problem, decision makers must set priorities among thousands of individual air pollutants as they formulate effective and efficient mitigation strategies.

**Objectives:**

Our aim was to compare and rank relative health risks of 179 air pollutants in Houston using an evidence-based approach supplemented by the expert judgment of a panel of academic scientists.

**Methods:**

Annual-average ambient concentrations by census tract were estimated from the U.S. Environmental Protection Agency’s National-scale Air Toxics Assessment and augmented with measured levels from the Houston monitoring network. Each substance was assigned to one of five risk categories (definite, probable, possible, unlikely, uncertain) based on how measured or monitored concentrations translated into comparative risk estimates. We used established unit risk estimates for carcinogens and/or chronic reference values for noncarcinogens to set thresholds for each category. Assignment to an initial risk category was adjusted, as necessary, based on expert judgment about the quality and quantity of information available.

**Results:**

Of the 179 substances examined, 12 (6.7%) were deemed definite risks, 9 (5.0%) probable risks, 24 (13.4%) possible risks, 16 (8.9%) unlikely risks, and 118 (65.9%) uncertain risks.

**Conclusions:**

Risk-based priority setting is an important step in the development of cost-effective solutions to Houston’s air pollution problem.

Despite three decades of progressively more extensive and stringent regulatory controls, there is ongoing concern about unhealthful ambient levels of air pollution in Houston, Texas (Mayor’s Task Force 2006; Rice University 2006). Houston, with a population of > 2 million, is the fourth largest city in the United States, and the 10-county Greater Houston region, with a population of > 5 million, is the nation’s seventh largest metropolitan area. Because Houstonians drive an average of > 140,000,000 miles/day, emissions from cars, trucks, and buses are a major source of airborne pollutants. Moreover, Houston is home to > 400 chemical manufacturing facilities, including two of the biggest refineries in the United States. The petrochemical complex along the Houston ship channel is the largest in the country, and the Port of Houston is the largest in the United States in terms of foreign tonnage and second largest in total tonnage. Aggregate airborne emissions from many small sources spread across Greater Houston, such as dry cleaners, gas stations, surface coating processes, and gasoline-fueled lawn maintenance equipment, add to the complex mixture of ambient air pollutants typically present in Houston’s air.

Meteorologic conditions and patterns also contribute to the air pollution problem in Houston. Between April and October there are usually a high number of warm sunny days with quiescent breezes, causing ground-level buildup of air pollutant concentrations. Most air pollution episodes in Houston occur as the wind direction rotates continuously over a 24-hr period, trapping a mass of unmoving air over the city. Elevated ambient levels of air pollution often occur along with high temperatures and humidity, creating hazy, malodorous, and oppressive conditions in the city.

Cost-effective mitigation of Houston’s air pollution predicament requires policy makers to set priorities among literally thousands of airborne compounds, and to make difficult trade-offs between the benefits of reducing risks to human health, on one hand, and the costs of controlling airborne emissions, on the other. At the outset, policy makers must decide, either implicitly or explicitly, which air pollution–related risks are the worst so that limited resources can be focused where they will do the most good. In this commentary we present a risk-based priority-setting process aimed at comparing and ranking relative human health risks of selected air pollutants known or suspected to be present in Houston’s air.

## Objectives

Despite documented air pollution problems in Houston, there has been no systematic effort to compare relative risks among the plethora of airborne chemicals that contribute to pollution-related health problems. Our goal was to gather available information on air pollution emissions and ambient concentrations in Greater Houston, and then to compare and rank chronic risks for Houstonians, using a procedure that combined quantitative risk estimates with scientific judgment. The analysis focused on 179 ambient air pollutants, including ozone and fine particulate matter (PM with aerodynamic diameter < 2.5 μm; PM_2.5_), for which National Ambient Air Quality Standards (NAAQSs) have been established, diesel particulates, which have been designated a “toxic air contaminant” by the State of California [California Air Resources Board (CARB) 1998], and 176 hazardous air pollutants (HAPs) from among those listed in the Clean Air Act (Clean Air Act Amendments 1990). The results could then be used to inform priorities for citywide monitoring and mitigation efforts.

## Methods

The process began with the recruitment of an expert panel, whose judgment would be relied on to both validate and refine the results of an analytical ranking procedure. The panel was comprised of eight academic specialists (two of the authors, K.S. and S.H.L., served as panel members, and the other three, D.M., H.B., and P.J.L., served as staff) from five local universities with a range of expertise, including toxicology, epidemiology, exposure assessment, risk analysis, occupational medicine, pediatric pulmonology, and chemical engineering. Their tasks were to review the relevant scientific evidence, examine available ambient concentration data, oversee the assignment of substances to specific risk-based categories, and then to use their collective judgment to refine these assignments as necessary.

**Figure 1:**
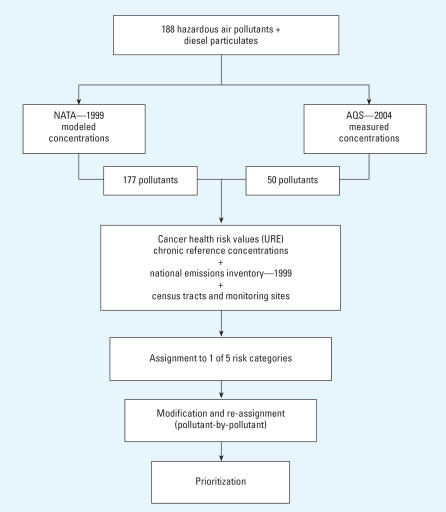
Flow chart of the risk ranking approach used to compare ambient air pollutants in Houston air. Abbreviations: AQS, air quality system; NATA, National-scale Air Toxics Assessment; URE, unit risk estimate.

Assignment of air pollutants to risk categories involved first setting category boundaries based on health-related toxicity values, and then using these values to impute concentration thresholds for each of the five categories. Measured and modeled concentrations for each chemical were compared with these thresholds to determine presumptive risk-category assignments. Chemicals were sorted twice within categories. The first sort was by percentiles of emission mass, relative to all chemicals inventoried, and the second was by the number of monitors or census tracts where concentrations exceeded the category threshold. Compounds near the upper or lower boundaries—for instance, those in a relatively low emissions percentile or those found in only a single location—were then considered for possible reassignment to a different category. The overall process is depicted schematically in Figure 1.

The approach shown in Figure 1 was applied to 177 of the 179 air pollutants. Ozone and particulate matter were treated differently, because they are “criteria” pollutants for which NAAQSs have been promulgated. Assignment of ozone to a particular risk category was based on how often, and by how much, ambient concentrations exceeded the NAAQS, whereas assignment of PM_2.5_ was based on whether levels either exceeded the old standard or were likely to exceed the new one. The task of assigning the HAPs to particular risk categories was more difficult by comparison for three reasons: There are currently no health-based standards; there tend to be fewer data on linkages between exposure and effects; and measurements of ambient concentrations are generally spotty or lacking completely.

To obtain estimates of ambient concentrations for as many HAPs as possible, modeled annual average concentrations for 1999 from the U.S. Environmental Protection Agency (EPA) National-scale Air Toxics Assessment (NATA) were used (U.S. EPA 2006a). Results from the NATA provided estimated ambient concentrations for 177 HAPs in 895 census tracts (each with approximately 4,000 inhabitants) in the 10-county Greater Houston area. The NATA values were derived by the U.S. EPA using a computerized air dispersion model that combined 1999 airborne emissions data from outdoor sources, including point, mobile (both on- and off-road), area, and background sources, with Houston-specific meteorologic variables. The model also took into consideration the breakdown, deposition, and transformation of pollutants in the atmosphere after their release. These modeled values were supplemented with measured 2004 annual concentrations for 50 substances (49 HAPs plus diesel particles) from 19 monitoring sites in and around Houston—14 in Harris County, 3 in Galveston, 1 in Brazoria, and 1 in Montgomery (U.S. EPA 2006b).

To get a sense of relative health risks associated with estimated ambient concentrations of HAPs, and to set category thresholds, we used health-related toxicity values developed by either the U.S. EPA or the California Office of Environmental Health Hazard Assessment (OEHHA), whichever was more stringent (health protective), (Cal EPA and OEHHA 2002; Cal OEHHA 2005; U.S. EPA 2005, 2006c, 2006d). In the few instances when no value was given by either U.S. EPA or California OEHHA, we used health values from other sources, such as the Agency for Toxic Substances and Disease Registry (ATSDR). For carcinogens, estimates were based on their respective unit risk estimates (UREs), which represent the excess lifetime cancer risk estimated to result from continuous lifetime exposure to an average concentration of 1 μg/m^3^ of a particular substance in air. For noncarcinogens, estimates were based on comparison of estimated ambient concentrations with chronic noncancer inhalation reference values, either reference concentrations (RfCs) used by the U.S. EPA, reference exposure levels (REL) used by California OEHHA, or minimum risk levels (MRL) used by ATSDR.

We assigned each HAP to a specific risk category based on how its measured or modeled annual-average concentrations compared with category thresholds computed from established UREs (carcinogens) and/or RfCs/ RELs/MRLs (noncarcinogens). Substances which there were no known emissions and/or inconsequential monitored or modeled concentrations were designated “unlikely risks” (suggestive evidence of negligible or insignificant risk to both the general population and vulnerable subgroups). Substances for which putative cancer risk was 1 ×10^−6^ to 1 ×10^−5^ and/or ambient concentrations were 5–75% of the applicable noncancer reference concentration (in at least one census tract) were deemed “possible risks” (partial or limited evidence that they might constitute a significant risk under certain circumstances). Substances that were estimated to represent a cancer risk between 1 ×10^−5^ to 1 ×10^−4^ and/or that were 76–100% of the relevant noncancer reference value (in at least one census tract) were labeled “probable risks” (substantial corroborating evidence that they are likely to represent significant risks under the right conditions). Substances for which attributed cancer risk was > 1 ×10^−4^ and/or ambient concentrations were > 100% of appropriate noncancer reference values (in at least one census tract) were classified as “definite risks” (compelling and convincing evidence of significant risk to the general population or vulnerable groups). Substances that posed a cancer risk of < 1 ×10^−6^ or whose ambient concentrations were < 50% of the RfC were deemed “uncertain risks” because there was inadequate or insufficient evidence to ascertain whether they posed a significant risk to Houstonians. The details of this process are illustrated for benzene in the Supplemental Material, Appendix 1 (available online at http://www.ehponline.org/docs/2007/10043/suppl.pdf)

Finally, initial risk-category assignments were adjusted, as mentioned earlier, based on relative emission quantities and number of census tracts or monitoring stations affected. Subsequent adjustments were made for 11 compounds: hydrazine (moved from definite to uncertain); nickel, manganese, and cadmium compounds (moved from definite to possible); acrylic acid (moved from definite to probable); vinyl chloride (moved from definite to probable); titanium tetrachloride (moved from probable to possible); 2,4-dinitrotoluene (moved from probable to possible); and 1,2-dichloropropane, ethyl acrylate, and quinoline (moved from possible to uncertain).

## Results

As shown in Table 1, 12 air pollutants were classified as “definite risks.” Because monitors in Greater Houston routinely surpass the ozone standard (0.08 ppm average for 8 hr), and have recorded some of the highest readings in the nation, ozone was characterized as a definite risk (respiratory, cardiovascular effects). Although Houston did not violate the NAAQS for PM_2.5_, ambient levels are near the standard and may exceed it in the not-too-distant future (U.S. EPA 2006e); therefore,ambient concentrations of PM_2.5_ were considered a definite health risk (respiratory, cardiopulmonary effects) for Houstonians. We also determined that airborne levels of seven carcinogens—diesel particulate matter, 1,3-butadiene, chromium VI, benzene, ethylene dibromide, formaldehyde, and acrylonitrile—pose an unacceptable increased cancer risk, that is, at least 1 theoretical excess cancer death for every 10,000 residents. In addition, it was concluded that five substances—1,3-butadiene (reproductive effects in addition to being a carcinogen), formaldehyde (respiratory effects and also a carcinogen), acrolein (respiratory effects), chlorine (respiratory effects), and hexamethylene diisocyanate (pulmonary and respiratory effects)—are present at ambient levels that represent an unacceptable increased risk for chronic (noncancer) disease.

In the context of this comparative risk exercise, certainty regarding assignment of a compound to a particular risk-based category was highest when modeled values were in close agreement with measured values. For 9 of 10 HAPs in the definite risk category, NATA-modeled concentrations exceeded the threshold conditions (cancer risk > 10^−4^ and/or ambient levels > 100% of RfC or REL), whereas the same was true for only 5 with monitored concentrations. In addition, total annual emissions of all 10 HAPs were well above the 50th percentile (mass) in the emissions inventory for 1999, and 9 (all except ethylene dibromide) were reported (either by NATA or the Air Quality Monitoring system) (U.S. EPA 2006b) in relatively high concentrations at multiple sites.

Two of the chemicals in the definite risk category, diesel particulate matter and chromium VI, cannot be measured directly. Computing their concentrations relied on the apportionment of measurements from other substances. For diesel particulates, we relied on a protocol from the California EPA using ambient concentrations of elemental carbon as a surrogate for estimating diesel particulate concentrations. Chromium VI involved a similar apportionment technique, relying on measures of chromium compounds. The details of these apportionments appear in the Supplemental Material, Appendix 2 (available online at http://www.ehponline.org/docs/2007/10043/suppl.pdf).

The evidence is not as strong but nevertheless persuasive that an additional nine air pollutants are likely to cause adverse health effects at concentrations measured or modeled in Houston air. These substances were designated “probable risks” (Table 2) and included eight carcinogens—vinyl chloride, acetaldehyde, ethylene dichloride, naphthalene, arsenic compounds, carbon tetrachloride, ethylene oxide, 1,1,2,2-tetrachloroethane—and one compound—acrylic acid—which has chronic noncancer effects.

The evidence available for another 24 air pollutants was even more limited, but still suggestive that Houstonians might, in certain situations, experience negative health consequences from exposure to plausible concentrations in ambient air. Twenty-two of these substances are carcinogens and, as summarized in Table 3, they were classified “possible risks.”

Sixteen air pollutants were categorized as “unlikely risks” because available evidence suggests they probably create no significant threat of harm for Houstonians. Of these 16 substances, two—coke oven emissions and nitrosodimethylamine—have zero reported emissions in Greater Houston, two (2,4-dinitrophenol,arsinine) have only negligible modeled ambient concentrations, and 12 have unknown emissions (1,3-propane sultone, 2,4,6-trichlorophenol, 2-chloroacetophenone, 3,3-dimethoxybenzidine, 3,3-dimethyl benzidine, chlorobenzilate, ethyl carbamate, ethylene thiourea, lindane, *N*-nitrosomorpholine, *p*-dimethylaminoazobenzene, toxaphene).

One hundred eighteen air pollutants were deemed to be “uncertain risks” (Supplemental Material, Appendix 3; available online at http://www.ehponline.org/docs/2007/10043/suppl.pdf) because there was inadequate or insufficient information to determine whether they currently pose a significant health threat to the residents of Houston. Of these 118 pollutants, 13 are not in the emissions inventory for Greater Houston, and of those that are, 16 are carcinogens for which only UREs are available, 45 are noncarcinogens for which only RfCs are available, 17 substances have both a URE and RfC (i.e., they are thought to have both carcinogenic and noncarcinogen effects), and 27 have neither a URE nor RfC.

## Discussion

Based on a survey of both monitored and modeled ambient concentrations, and comparison of ambient levels with health-based thresholds, each of 179 air pollutants was assigned to one of five relative-risk categories based on the likelihood that it posed a chronic health threat to Houston residents. Of the 179 substances examined, 12 (6.7%) were deemed definite risks, 9 (5.0%) probable risks, 24 (13.4%) possible risks, 16 (8.9%) unlikely risks, and 118 (65.9%) uncertain risks. Pollutants in the definite-, probable-, and possible-risk categories represent a mix of carcinogens and noncarcinogens emitted by a diversity of area, mobile, and stationary sources.

The approach taken here fits into a broader context of methods known collectively as comparative risk assessment (Carnegie Commission 1993; Davies 1996; Sexton 1999). Comparative risk assessment is a tool for systematically organizing and analyzing information about disparate environmental problems in a way that allows for comparison of relative risks (i.e., distinctions according to probability of occurrence and magnitude/gravity of effects) and identification of the most serious threats to health (i.e., recognition of most likely and most harmful). It is both an analytical process and a set of related techniques that use available data in conjunction with expert judgment to compare and ultimately prioritize a spectrum of environmental health problems. Comparative risk assessment is broader, more uncertain, and less well defined than traditional quantitative risk assessment, and it is hampered by scarcity of relevant data, limited scientific understanding, lack of suitable methods, and absence of formal guidelines spelling out default assumptions and exposure scenarios. Consequently, even in its most scientific form, comparative risk assessment is necessarily an exercise in professional judgment based on uncertain estimates of divergent risks (Sexton 1999).

Yet despite these inherent limitations, comparative risk assessment has emerged as an important decision-making tool used by federal, state, and local authorities to help set risk-based priorities. Starting in the early 1980s, the U.S. EPA began using this instrument to rank diverse environmental risks for the purpose of establishing management and resource directions. By the mid-1990s, all 10 U.S. EPA regions and numerous states, cities, and tribes had conducted locality-specific comparative risk assessments (Sexton 1999). Today, comparative risk assessment provides decision makers with a unifying conceptual framework and a common language to evaluate, compare, and rank environmental hazards. When combined with analyses of cost for risk-reduction strategies, results of comparative risk assessments can aid in identifying cost-effective policy options.

Urban air pollution is a complicated concoction of gases, liquids, and particles comprising thousands of individual substances, and several studies have attempted to compare relative risks among selected chemical constituents in defined locations (Caldwell et al. 1998; Fox ex al. 2004; Morello-Frosch et al. 2000; Tam and Neumann 2004). Using generally similar methods and approaches, such as employing ambient monitoring data or modeled concentrations to estimate exposure and relying on established reference values from government agencies to compare chronic health risks, these studies demonstrated that ambient levels of numerous urban air pollutants commonly exceed health-related benchmarks. Building on this methodology, we combined modeled and measured estimates, considered emissions inventory and location data, and added an expert judgment component to assign substances to one of five risk-based categories. These risk categories provided a readily accessible means of organizing complex data to compare and rank relative air pollution–related health risks for Houstonians.

### Caveats

By official count, there are > 60 air pollution monitors operating at 39 locations and screening for > 130 chemicals in the Greater Houston area [Texas Commission on Environmental Quality (TCEQ), personal communication]. It has been said that “[t]he air quality in Houston is monitored more closely and analyzed with more intensity than perhaps anywhere in the country—if not the world” (TCEQ 2005). Nonetheless, estimating ambient exposures was problematic because active monitors supplying data to the air quality system (U.S. EPA 2006b) are located in just 19 of 895 census tracts, are not spread evenly across the 10-county Greater Houston area (most are near the Houston ship channel), and measure only 50 of the 177 hazardous air pollutants examined in this study. Further, few pollutants were measured consistently over time or in all 20 locations; for example, benzene was measured at 15 public sites in 1999 and 16 in 2004. Accordingly, for ambient exposure estimates we depended heavily on the NATA, which used data from the 1999 National Emission Inventory in combination with the ASPEN (Assessment System for Population Exposure Nationwide) model (U.S. EPA 2006a) to estimate annual average air pollutant concentrations by census tract. Although the NATA is based on sound scientific principles and uses best available data on emissions and meteorology, results necessarily depend on numerous assumptions and postulations that have not been verified.

There is evidence (U.S. EPA 2007) that the NATA may underestimate actual monitored concentrations for some compounds, including metals such as chromium, lead, manganese, and nickel (underestimated by ≥ 75%) and volatile organic compounds such as acetaldehyde, benzene, and formaldehyde (underestimated by ≥ 50%). It is also possible that risks from noncarcinogens are underestimated by the ranking scheme. Castorina and Woodruff (2003), for example, estimated that about half of the RfCs they analyzed would translate into lifetime risks of 1 ×10^−3^ assuming a linear dose–response relationship. Furthermore, even if noncarcinogens have putative thresholds, the combined effects from exposure to multiple HAPs with common health end points are likely to raise the cumulative dose response above any practical threshold levels (Clewell and Crump 2005).

The expert judgment component of the ranking scheme was informal and relied on the collective opinion of a scientific panel to refine initial risk-category assignments. A more prescribed procedure could have been used to incorporate qualitative information into the assessment, such as assigning a higher risk designation to reproductive and developmental toxicants or to compounds that persist and bioaccumulate in the environment (Lunder et al. 2004).

Results of this comparative risk assessment must, therefore, be interpreted with care. In general, efforts to measure air pollution–related risks (both morbidity and mortality) directly are stymied by an array of problems that make it difficult to establish causality between typical levels of urban air pollution and observed adverse health effects. Among the common obstacles that normally confront assessments of environmental health risks are the following: incomplete understanding of disease etiology; wide range of nonenvironmental causes for most diseases to which environmental agents contribute; environmental pollutants that often enhance or exacerbate, rather than cause disease or dysfunction; lack of suitable methods, measurements, and models to *a*) estimate exposure, dose, and effects, and *b*) characterize variability over individuals, time, and space; deficiency of surveillance and reporting systems for exposure and environmentally related health outcomes; long latency period from exposure to negative health consequences for many environmentally induced diseases (e.g., lung cancer); real-world exposures occurring not to a single pollutant, but to complicated mixtures of environmental agents that vary both temporally and spatially; observed health end points (e.g., lung damage) that may not be the primary target of the environmental agent (e.g., immune system); and inherent variability among individuals in terms of biological (e.g., genetic) susceptibility to environmentally induced illness and injury.

In this study we considered only a specific and narrowly defined type of risk—namely the harmful chronic (long-term) effects of human inhalation exposure to estimated annual average outdoor concentrations of 179 chemical substances. Peak concentrations from accidental chemical releases and routine maintenance at chemical plants can have acute (short-term) effects on people, as well as cause serious impairment to ecologic resources (e.g., fish, wildlife) and damage to social welfare (e.g., poor visibility, degraded property values). People are exposed to other chemical, biological, and physical agents in the air they breathe, and real-life exposures are caused not just by outdoor air pollutants but also airborne contaminants inside residences, cars, workplaces, restaurants, and other settings. Consideration of these and other potentially noteworthy factors, such as cumulative effects from simultaneous or sequential exposure to multiple stressors by multiple pathways and routes, were explicitly excluded from this assessment to make the task practical and manageable within time and resource constraints.

### Challenges

The identification of ozone, PM_2.5_, and diesel exhaust as definite health risks was relatively straightforward owing to the comparatively large database on adverse health effects that exists for each substance, along with clear evidence that people are exposed to outdoor levels considered unsafe. The picture was generally less certain and more problematic for the HAPs, which include a diverse mix of carcinogens and systemic toxicants. These air pollutants historically have received less regulatory attention, and ambient concentrations and exposure-effect relationships tend to be less well characterized. Unambiguous assignment of these substances to a particular risk category is often hindered by incomplete and inadequate data, making it necessary in many instances to use scientific judgment as a basis for extrapolating beyond the limited or nonexistent database.

The intrinsic challenges of comparing HAPs-related health risks are illustrated by the fact that 118 (66%) of the substances examined by the task force were assigned to the uncertain risk category. This decision was based on the panel’s collective judgment that there was insufficient evidence on hand to ascertain whether these substances currently pose a significant threat to the health and well-being of Houston residents. In short, it was not possible to say with an acceptable degree of certainty whether these compounds are a health risk or not. From a public health perspective this obviously leaves us in an unsatisfying situation, wherein we lack the necessary scientific information to distinguish among definite, probable, possible, and unlikely health risks.

It must be remembered that relatively few resources (< $30,000) and only limited time (approximately 18 months) were devoted to this project. It is likely that additional resources and a longer project period could have reduced the number of HAPs assigned to the uncertain risk category. In the end, however, only targeted research and improved surveillance will allow us eventually to *a*) determine the appropriate risk category for many HAPs presently listed as uncertain risks, and *b*) verify the risk assignments for HAPs in other categories.

## Conclusions

Substantial efforts have been devoted over the years to scrutinizing air pollution levels in Houston, and considerable resources have been expended on mitigation measures. Although the success of these endeavors is difficult to quantify, it appears that levels of some air pollutants, like ozone, have decreased since the early 1980s even though Houston’s population, economy, and traffic have grown steadily. Much of the progress over the past 35 years can be attributed to regulatory controls mandated by the 1970 Clean Air Act and subsequent amendments (1990). But air quality improvements in Houston appear to have slowed or even stalled since about 2000, and there is legitimate concern that matters will only get worse. A critical first step in finding cost-effective solutions is to identify those airborne pollutants that represent the most serious health risks so that control strategies can be designed to focus on the worst risks first.

As part of the search for cost-effective solutions, we must acknowledge that air pollution is a by-product of our culture and our way of life. It is produced as a direct result of choices we make, both individually and collectively, about energy sources, technologies, economic activities, and lifestyles. Although the relative contribution of a particular source or source category may vary from place to place, it is the blending together of combined emissions from numerous point, mobile, and area sources that makes Houston’s air quality unhealthful. Thus, focusing control efforts exclusively on a single type of source, no matter how obvious or obnoxious, is unlikely, by itself, to solve the problem.

Comparative risk assessment is a “decision tool” for organizing and analyzing information about air pollution in a manner that will aid decision makers as they choose among competing priorities. It is not, in our opinion, a decision rule that automatically and inevitably leads to a specific conclusion about resource allocation. We hope these risk rankings will be a useful adjunct to other relevant information, and that results will contribute to informed decisions not only about how to use available resources more effectively and efficiently, but also about how to justify the need for additional funding.

## Correction

Table 4, published online in the original manuscript, has been deleted here. The relevant information now appears in “Results.”

## Figures and Tables

**Table 1:** Basis and data source for classifying air pollutants in “definite risks” category in Greater Houston.^a^

				Data source				
				AQS 2004^b^			NATA 1999^c^	
	Basis			No. of monitors			No. of census tracts	
Air pollutant	Cancer risk	Chronic risk	NAAQS criteria exceedance	Cancer risk	Chronic risk	Days in exceedance	Cancer risk	Chronic risk
Ozone			X		20	46		
PM_2.5_			X			0		
Diesel PM	X	X		6^d^			895	43
1,3-Butadiene	X	X		7	1		9	1
Chromium VI	X			2^e^			433	
Benzene	X			2			66	
Ethylene dibromide	X			1				
Acrylonitrile	X						6	
Formaldehyde		X			2			143
Acrolein		X			3			889
Chlorine		X						31
1,6-Hexamethylene Diisocyanate		X						6

Abbreviations: AQS, Air Quality System; X denotes the basis for risk classification.

a Greater Houston consists of the 10-county, Houston–Sugar Land–Baytown metropolitan statistical area defined by the U.S. Census Bureau (2003).

b Data from U.S. EPA (2006b).

c Data from U.S. EPA (2006a).

d Diesel estimated using measured ambient elemental carbon concentrations.

e Chromium VI estimated using measured ambient chromium PM_2.5_ concentrations; see Supplemental Material, Appendix 3 (available online at http://www.ehponline.org/docs/2007/10043/suppl.pdf).

**Table 2:** Basis and data source for classifying air pollutants in “probable risks” category in Greater Houston.^a^

			Data source			
			AQS 2004^b^		NATA 1999^c^	
	Basis		No. of monitors		No. of census tracts	
Air pollutant	Cancer risk	Chronic risk	Cancer risk	Chronic risk	Cancer risk	Chronic risk
Vinyl chloride	X				1	
Acrylic acid		X				1
Acetaldehyde	X	X	2	1	48	1
Ethylene dichloride(1,2-dichloroethane)	X		1		5	
Naphthalene	X				10	
Arsenic compounds(inorganic may include arsine)	X				7	
Carbon tetrachloride	X		16		895	
Ethylene oxide	X				9	
1,1,2,2-Tetrachloroethane	X		2			

Abbreviations: AQS, Air Quality System; X denotes the basis for risk classification.

a Greater Houston consists of the 10-county, Houston–Sugar Land–Baytown metropolitan statistical area defined by the U.S. Census Bureau (2003).

b Data from U.S. EPA (2006b).

c Data from U.S. EPA (2006a).

**Table 3:** Basis and data source for classifying air pollutants in “possible risks” category in Greater Houston.^a^

			Data source			
			AQS 2004^b^		NATA 1999^c^	
	Basis		No. of monitors		No. of census tracts	
Air pollutant	Cancer risk	Chronic risk	Cancer risk	Chronic risk	Cancer risk	Chronic risk
Nickel compounds	X	X			1	1
Manganese compounds		X				1
Cadmium compounds	X	X	6		2	1
Titanium tetrachloride		X				1
2,4-Dinitrotoluene	X				1	
Methyl *tert*-butyl ether	X		1		61	
1,3-Dichloropropene	X				9	
Chloroform	X		16		41	
Methylene chloride	X				56	
(dichloromethane)						
p-Dichlorobenzene	X				64	
Propylene oxide	X				8	
Tetrachloroethylene	X		16		683	
(perchloroethylene)						
Trichloroethylene	X				2	
1,1,2-Trichloroethane	X		16			
Bis(2-ethylhexyl)phthalate	X				895	
Epichlorohydrin	X				3	
(1-chloro-2,3-epoxypropane)						
Lead compounds	X				1	
1,2-Dibromo-3-chloropropane	X				3	
1,4-Dioxane	X				2	
2,4-Toluenediamine	X				1	
Acrylamide	X				1	
Benzidine	X				2	
Dichloroethyl ether	X				1	
Bis(2-chloroethyl)ether						
Polycyclic organic matter	X				76	

Abbreviations: AQS, Air Quality System; X denotes the basis for risk classification.

a Greater Houston consists of the 10-county, Houston–Sugar Land–Baytown metropolitan statistical area defined by the U.S. Census Bureau (2003).

b Data from U.S. EPA (2006b).

c Data from U.S. EPA (2006a).
